# Poly(3‐Hydroxybutyrate‐*Co*‐3‐Hydroxyhexanoate): Real‐Time Monitoring of Microbial Degradation via Quartz Crystal Microbalance and Electrochemical Measurement

**DOI:** 10.1002/bip.70044

**Published:** 2025-09-25

**Authors:** Noriyuki Asakura, Takuma Otsuki, Momoko Kitamura, Tomohiro Hiraishi, Hideki Abe

**Affiliations:** ^1^ Bioplastic Research Team RIKEN Center for Sustainable Resource Science (CSRS) Wako‐shi Saitama Japan; ^2^ School of Life Science and Technology, Institute of Science Tokyo Meguro‐ku Tokyo Japan

**Keywords:** cyclic voltammetry (CV), impedance measurement, poly(3‐hydroxybutyrate‐*co*‐3‐hydroxyhexanoate) (PHBH), quartz crystal microbalance (QCM), scanning electrochemical microscopy (SECM)

## Abstract

Poly(3‐hydroxybutyrate‐*co*‐3‐hydroxyhexanoate) (PHBH), produced by some bacteria, including *Aeromonas* strains, exhibits excellent environmental biodegradability, even in marine environments where biodegradation is typically poor. However, the exact mechanisms underlying this biodegradability remain to be elucidated. To evaluate the mechanisms of microbial degradation of PHBH, focusing on the initial stages, PHBH degradation by *Comamonas testosteroni* is analyzed, using a quartz crystal microbalance (QCM), cyclic voltammetry (CV), impedance, and scanning electrochemical microscopy (SECM). Real‐time monitoring of bacterial adsorption followed by PHBH degradation is quantitatively achieved at the cellular level using a highly sensitive QCM. CV and impedance measurements suggest that microbial degradation of PHBH proceeds in a heterogeneous manner. The SECM observations reveal the heterogeneous microbial degradation of PHBH, which is highly consistent with the QCM, CV, and impedance measurements. These findings indicate that this analytical system, combined with highly sensitive QCM analysis and electrochemical measurement, is an effective tool for studying the microbial degradation of biodegradable plastics.

## Graphical abstract

Bacteria adsorb to PHBH film within hours and degrade it in a heterogeneous manner, revealed by highly sensitive QCM and electrochemical measurements.

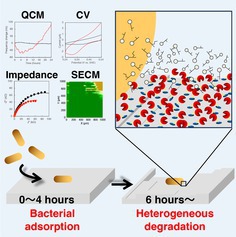

## 1 Introduction

Plastics, which have become indispensable in daily life, are widely used in many industries, including food and medical packaging, construction, automotive parts, and the agricultural sector, owing to their ease of processing, durability, low cost, and availability [1, 2]. However, as plastics persist even under harsh environmental conditions, the accumulation of plastic pollution has become one of the most pressing environmental issues. Discarded plastics are considered one of the main causes of marine environmental pollution [3]. Novel plastic materials that can reduce the risk of plastic waste pollution are therefore required.

Biomass‐based biodegradable plastics that can be produced via mineralization by microorganisms are emerging as promising candidates to address this issue [4]. These include poly(hydroxyalkanoate) (PHA), which is synthesized from biomass by microorganisms and degraded and assimilated by PHA‐degrading microorganisms. PHAs typically exhibit biocompatibility, water insolubility, gas‐barrier properties, low toxicity, and low viscosity when melted [5]. PHAs may therefore be suitable for household, agricultural, industrial, and medical applications. Poly(3‐hydroxybutyrate‐*co*‐3‐hydroxyhexanoate) (PHBH), a type of PHA, has a tailor‐made composition consisting of both highly crystalline (3‐hydroxybutyrate [3HB]) and elastomeric (3‐hydroxyhexanoate [3HH]) units, giving it excellent thermal stability, promising mechanical performance, and a wide processing window. Increasing the 3HH content reduces its crystallinity and lowers its melting temperature, thereby expanding the processing window with minimal thermal degradation. Consequently, PHBH may be employed in applications where both flexibility and room temperature composting are required. When plastics are used in natural environments, such as in agriculture, there is a high risk of them contaminating the environment during and after use. Environmental biodegradability of plastics, particularly in the marine environments where they eventually accumulate, is therefore an important aspect of their implementation in terms of the implications for society. In Japan, PHBH is considered a pioneer material for marine biodegradable bioplastics, having received “OK Biodegradable MARINE” certification in September 2017, and is being developed for single‐use plastic applications such as in food packaging containers, plastic bags, and straws [6]. Relative to other non‐PHA bioplastics, PHBH has excellent biodegradability, even in the surface and deep seawaters where biodegradation is typically poor [7]. Nonetheless, the mechanisms of this biodegradability, especially in marine environments, remain to be elucidated.

The biodegradability of plastics has been examined in the laboratory via several conventional methods [8, 9]. First, changes in the mass and surface characteristics of plastic materials can be monitored after their in situ biodegradation. Second, biochemical oxygen demand (BOD) can be measured during burying or immersing plastics in environmental samples. Third, changes in the mass, surface characteristics, and physical properties of the plastics can be examined after burying or immersing them in the collected environmental samples for a specific period. These methods, however, can only indirectly reveal the mechanisms of biodegradation processes.

More specifically, bacterial degradation of biodegradable plastic is typically evaluated based on the size of the clear zone around the plastic‐dispersing agar medium or the change in the mass and surface properties of the plastic after immersion in a bacterial suspension or environmental sample. Conventional measurement of mass requires relatively few resources, although it lacks accuracy in detecting small changes in mass. Samples of degraded plastic must be pre‐treated before weighing, which complicates the measurement process and risks altering the mass of the sample. Atomic force microscopy (AFM) and scanning electron microscopy (SEM) are commonly used for surface analysis of biodegraded plastic materials, including PHBH [10, 11, 12]. AFM can generate detailed data on the surface topography of plastic and the changes occurring in plastic materials, while SEM can reveal the physical deterioration of plastic surfaces resulting from environmental or microbial exposure. In general, although these methods have high spatial resolution, they offer low temporal resolution. Using SEM and AFM, it is therefore difficult to quantify the heterogeneous degradation of plastics at cellular levels in real time; consequently, these methods provide limited information about the early stages of microbial plastic degradation. Furthermore, these methods require sample preparation and tend to involve the use of large, complex equipment.

The biodegradation process of biodegradable plastic is assumed to proceed as follows: degrading bacteria are adsorbed onto the plastic surface; the bacteria then secrete enzymes, which cause the plastic to degrade; the bacteria then consume and metabolize the degradation products, and a bacterial colony forms; finally, a biofilm forms. To elucidate the mechanisms of biodegradable plastic degradation, it is especially important to investigate the initial processes of degradation (bacterial adsorption, enzyme secretion, and plastic degradation), which are difficult to assess using conventional methods. Therefore, direct and real‐time evaluation of microbial behavior during the initial stages of biodegradation is essential.

The quartz crystal microbalance (QCM) is a sensitive tool for kinetic analysis of protein adsorption on polymer films and of enzymatic polymer degradation [13, 14, 15, 16, 17, 18, 19, 20, 21, 22]. The QCM is based on the piezoelectric nature of a quartz crystal; the change in mass is electrically detected as a shift in the resonant frequency, with sensitivity on the scale of ng cm^−2^ [23]. Thus, QCMs enable real‐time monitoring of the minimal loss of mass associated with polymer degradation. Nonetheless, conventional QCM is only accurate to ca. 5 ng, which is insufficient to examine mass gain or loss at the cellular level. Furthermore, using a QCM alone, it is difficult to determine whether polymer degradation proceeds in a homogeneous or heterogeneous manner.

Electrochemical techniques have attracted considerable attention as tools for assessing the surface states of materials. The electrochemical QCM (EQCM) combines a QCM and cyclic voltammetry (CV), thus measuring the current at the substrate's surface simultaneously with the change in mass [24, 25, 26]. This makes it possible to distinguish between homogeneous or heterogeneous degradation of a nonconductive polymer film on the QCM substrate by monitoring changes in current caused by the partial exposure of the gold substrate during degradation. In the electrochemical impedance method, an AC voltage is applied to the electrodes, and the impedance associated with the resulting AC current is obtained. In this process, an electric circuit model comprising a charging layer and a resistive layer can be constructed by analyzing the impedance when an AC voltage is applied at different frequencies. This method has been used to analyze phenomena analogous to the degradation of biodegradable plastics, such as the corrosion of painted metals and degradation of polymers [26, 27, 28, 29]. Finally, scanning electrochemical microscopy (SECM) is a promising tool for visually analyzing polymer surfaces at high resolution [30, 31]. SECM scans a sample surface using a microprobe electrode and can detect reactions on the surface, thereby providing localized information on surface properties at submicrometer scales. SECM imaging enables the quantification of chemical reaction properties and their localization on polymeric surfaces, including those composed of nonconductive polymers.

To address the need for direct and real‐time evaluation of microbial behavior during the initial stages of biodegradation, we constructed a system for real‐time high‐sensitivity monitoring of the microbial degradation of PHBH bioplastics, examining both microbial adsorption on the plastic surface and the subsequent degradation. This system can simultaneously evaluate homogeneous and heterogeneous degradation. To achieve this, a highly sensitive QCM system [24, 25], which can detect bacterial‐cell level changes in mass, was used to measure degradation. CV, impedance, and SECM measurements were performed to investigate whether the microbial degradation of PHBH was homogeneous or heterogeneous.

## 2 Results and Discussion

### 2.1 EQCM Measurement of PHBH Microbial Degradation

**FIGURE 1:**
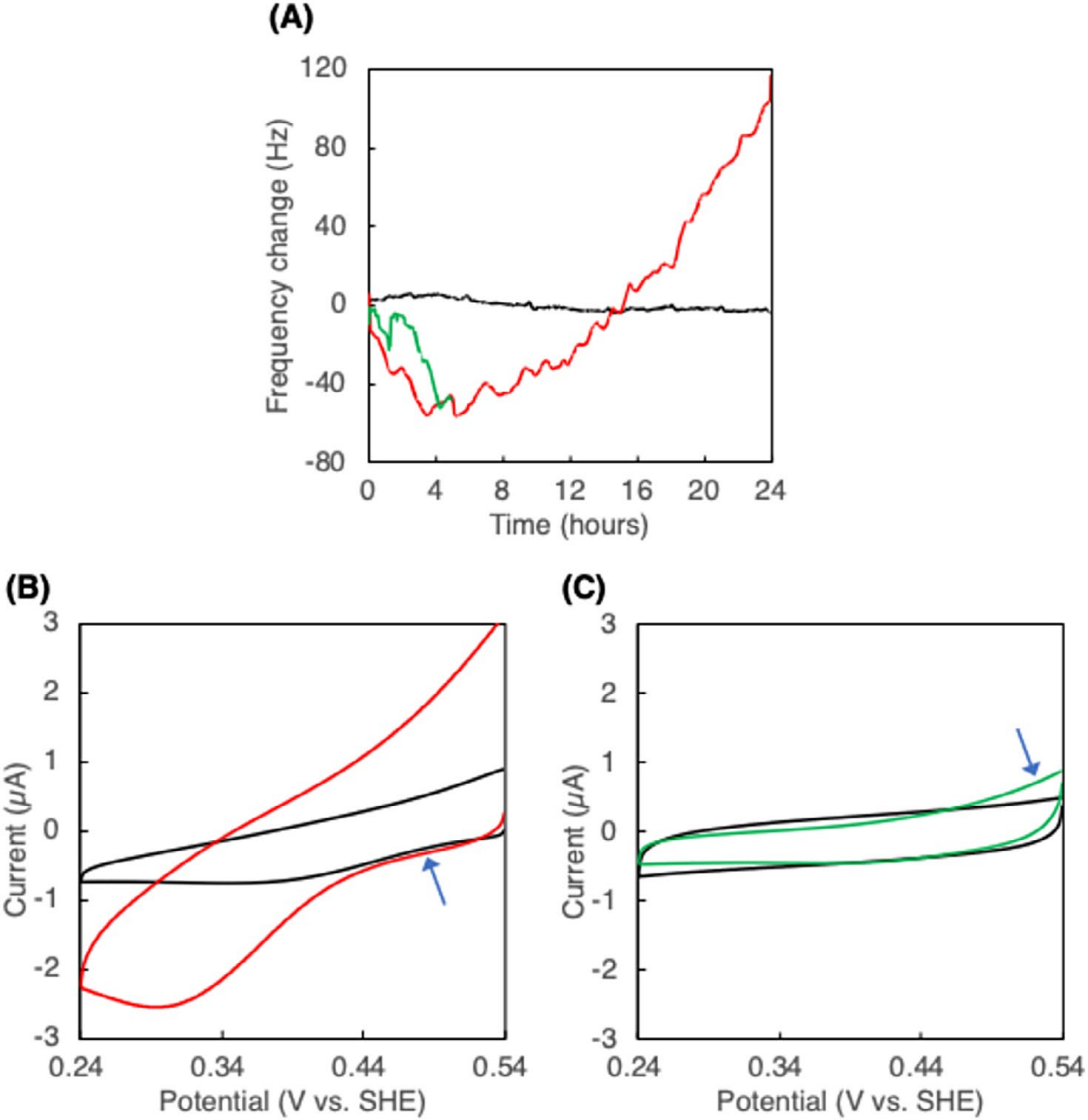
(A) Time courses of QCM frequency changes of PHBH‐fabricated electrode with (red: 24 h degradation; green: 5 h degradation) and without *Comamonas testosteroni* (black). (B) CVs of PHBH‐fabricated electrode before (black) and after 24 h degradation (red). (C) CVs of PHBH‐fabricated electrode before (black) and after 5 h degradation (green).

A PHBH film (2000 ng) was prepared on the QCM substrate. PHBH microbial degradation by *Comamonas testosteroni* was conducted at 34°C and monitored in real‐time via the highly sensitive QCM, in which water vibration in the monitoring cell is suppressed using the surface tension of water and the noise from the alternating current arising from the quartz crystal shear wave is blocked using a shield [24, 25]. Figure 1A presents the changes in frequency as a function of time during this microbial degradation (red line). A decrease in frequency occurred immediately after the injection of the bacterial suspension; the decline in frequency continued until approximately 4 h later, by which time the frequency had declined by ca. 56 Hz, suggesting an increase of about 75 ng, according to the Sauerbrey equation. Reports indicate that bacterial cell number and frequency shift are linearly related, and that bacterial cell mass accumulation can be described by the Sauerbrey equation [32, 33, 34]. Thus, this decline in frequency indicates that the mass on the QCM substrate surface gradually increased with incubation time, indicating bacterial adsorption onto the PHBH film surface over time. Assuming that the bacterium has short and long diameters of 1 and 2 μm, respectively, with a cylindrical shape with hemispheres at both ends, using a suspended density of ca. 1.1 g cm^−3^ [35], the mass of one bacterial cell was calculated to be 1.44 pg. Accordingly, it was estimated that ca. 52,000 bacterial cells were adsorbed onto the surface of the PHBH film (0.196 cm^2^) within 4 h after the addition of the bacterial suspension. This suggests that the adsorbed bacterial cells covered 0.47% of the surface area of the PHBH film. The frequency began to increase 6 h after the addition of the degrading bacteria, with the increase reaching 104 Hz after 24 h. This increase reflects a decline in the substrate surface mass, indicating PHBH film degradation. From 6 to 24 h after the start of the reaction, 214.4 ng (corresponding to a frequency increase of 160 Hz) of PHBH film (ca. 10% of the total film) was degraded by *C. testosteroni*. Between 14 and 24 h, the change in frequency with PHBH degradation was linear. Based on these findings, and assuming limited cell adsorption and growth during this period, it was estimated that *C. testosteroni* degraded the PHBH film at a rate of 0.302 pg h^−1^ cell^−1^ under the experimental conditions.

To evaluate the microbial degradation process, electrochemical measurements (CV) were performed on the PHBH film electrode after 24 h of degradation, and the post‐degradation voltammogram (red line) was compared to that before degradation (black line, Figure 1B): the charge detected at ca. 0.5 V was not significantly altered by degradation (indicated by an arrow in Figure 1B), suggesting that the thickness of the PHBH film on the electrode was not substantially affected by degradation. In contrast, the post‐degradation voltammogram reveals a significant increase in anode current at the positive potential region > 0.5 V and a peak current at the cathode ca. 0.3 V. The cathode current peak value observed in the post‐degradation voltammogram was consistent with the current observed for the unmodified gold electrode, at which the current was derived from the adsorption of OH^−^ and H^+^, suggesting that PHBH degradation exposed the gold surface beneath the PHBH film. This indicates that the microbial degradation of the PHBH film did not significantly alter the overall thickness of the film; however, the film on the electrode was partially degraded, resulting in the localized exposure of the gold surface and subsequent increase in conductivity.

From 4 to 6 h after the addition of the bacterial suspension, the frequency remained constant (red line, Figure 1A). During microbial degradation of bioplastics, adsorption of bacteria onto the plastic surface is thought to be followed by plastic degradation. Thus, the apparently constant mass during this period may indicate that, while bacterial adsorption was completed, PHBH degradation had not started, or that bacterial adsorption and PHBH degradation occurred simultaneously. To better understand the degradation phenomena that occurred during this period of constant frequency, we performed QCM monitoring until 5 h after the addition of the bacteria (green line, Figure 1A), as well as measured the CV of the PHBH film‐fabricated electrode after 5 h of microbial degradation (green line, Figure 1C). The QCM‐based curve of time‐dependent change in frequency (green line) was similar to the red line in Figure 1A, with the frequency remaining constant between 4 and 5 h after the start of the reaction. The CV (green line, Figure 1C) at 5 h after the addition of the bacteria reveals a marginal increase in anode current at ca. 0.5 V (indicated by an arrow in Figure 1C) relative to that before degradation (black line), suggesting that the PHBH film was slightly degraded at this time. Thus, these results indicate that PHA depolymerase expression and secretion were induced during the late stage of bacterial adsorption, followed by PHBH degradation by the secreted enzyme.

### 2.2 Impedance Measurement to Assess PHBH Microbial Degradation

**FIGURE 2:**
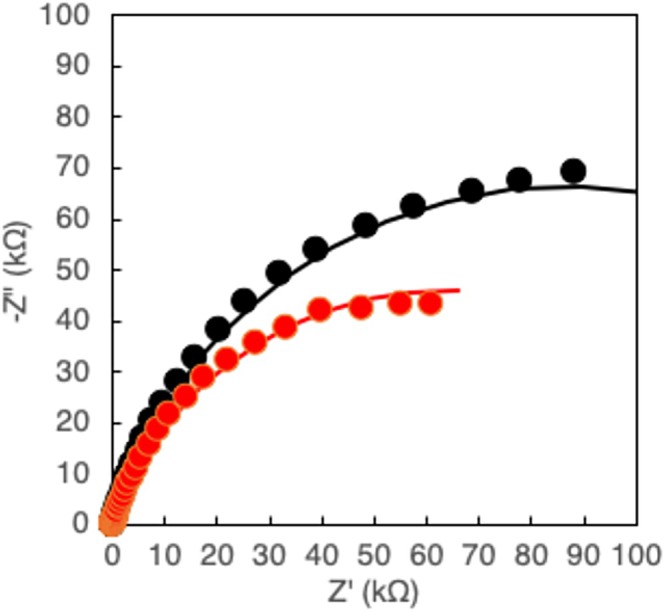
Nyquist plots of PHBH film (2751 ng) formed electrode before (black circles) and after 24 h degradation (red circles) with their fitting curves.

A PHBH film (2751 ng) was prepared on the electrode and subjected to degradation for 24 h in mineral medium at 34°C with a bacterial concentration of 2.4 × 10^8^ cells mL^−1^. After 24 h of degradation, the mass of the film was determined to be 1700 ng, and the impedance of the electrode after degradation was measured and plotted in Nyquist form (Figure 2). The pre‐degradation (black circles) and post‐degradation (red circles) plots are semi‐elliptical, with the major axis on the horizontal axis. Impedance was lower after degradation than before degradation.

### 2.3 Construction of Equivalent Circuit Model and Parameter Estimation

**FIGURE 3:**
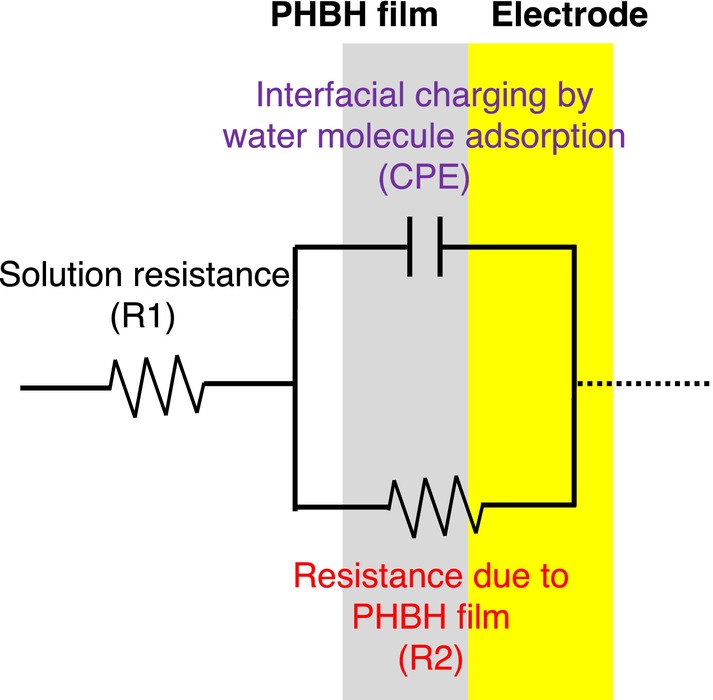
An equivalent circuit model used for the impedance analysis of PHBH film system.

The semi‐elliptical shape of the Nyquist plots reflects the nonuniformity of the electrode surface capacitance, which may indicate an uneven electrode surface, adsorption of substances on the electrode, or coating of the electrode with paint [36, 37, 38, 39, 40]. Based on the impedance data, a constant phase element (CPE) was then used to construct an equivalent circuit model [41, 42] of the microbial degradation of PHBH (Figure 3). CPE was introduced to improve the model fit, which was suboptimal owing to the nonideal capacitance behavior. In this model, *R*_1_ refers to the solution resistance, *R*_2_ (charge transfer resistance) refers to the resistance of the PHBH film, and CPE refers to the charge or discharge due to the adsorption or desorption of water molecules on the electrode. The impedance (*Z*) of this circuit is expressed as:

(1) $$Z=Z_{R1}+Z_{R2}Z_{\mathrm{CPE}}/\left(Z_{R2}+Z_{\mathrm{CPE}}\right)=Z^{\prime }-{\mathrm{jZ}}^{\prime \prime }$$

where *Z*_*R*1_ and *Z*_*R*2_ are the impedances of resistors *R*_1_ and *R*_2_, respectively; *Z*_CPE_ is the impedance of CPE, and *Z*′ and *Z*″ are the real and imaginary parts, respectively. The impedance *Z*_CPE_ of the CPE is expressed as

(2) $$Z_{\mathrm{CPE}}=1/\left\{{\left(\mathrm{j\omega}\right)}^{n}Q\right\}$$

where *Q* is the CPE constant, *j* is the imaginary number, *ω* is the angular frequency, and *n* is a value indicating the deviation from purely capacitive behavior (0 ≤ *n* ≤ 1) [43, 44, 45]. Here, the closer *n* is to 0, the rougher the surface is. Therefore, during the microbial degradation of the PHBH film, the roughness of the film surface after degradation can be evaluated by determining the *n* value.

We measured the impedance of electrodes fabricated with PHBH films of different thicknesses and performed equivalent circuit fitting on the obtained plots using an EIS Spectrum Analyzer. The Nyquist plots were fitted with good accuracy (Table 1). The *R*_1_ values range from a few ohms to several tens of ohms and were less than 1/1000 of the *R*_2_ values. As the same electrolyte solution was used in all impedance measurements, the solution resistance *R*_1_ can be regarded as nearly constant and negligible relative to *R*_2_. The post‐degradation *R*_1_ value (14.9 Ω) was almost the same as the pre‐degradation value (14.8 Ω), indicating that the solution resistance was not affected by degradation.

**TABLE 1:** Quantitative parameters estimated from the experimental impedance data for poly(3‐hydroxybutyrate‐*co*‐3‐hydroxyhexanoate) (PHBH) film electrodes using a constant phase element (CPE) model of the equivalent circuit.

Substrate	*R*_1_ (Ω)	*R*_2_ (kΩ)	*Q* (μF)	*n*
PHBH 0 ng (film unformed)	6.18	19.2	18.7	0.912
PHBH 100 ng	12.0	44.1	12.8	0.876
PHBH 555 ng	14.0	65.8	11.7	0.874
PHBH 587 ng	32.8	79.1	11.4	0.875
PHBH 1686 ng	6.92	116.2	7.31	0.865
PHBH 2226 ng	17.3	141.7	6.46	0.834
PHBH 2751 ng	14.8	174.1	6.55	0.830
PHBH 1700 ng (after degradation)	14.9	127.3	10.2	0.798

**FIGURE 4:**
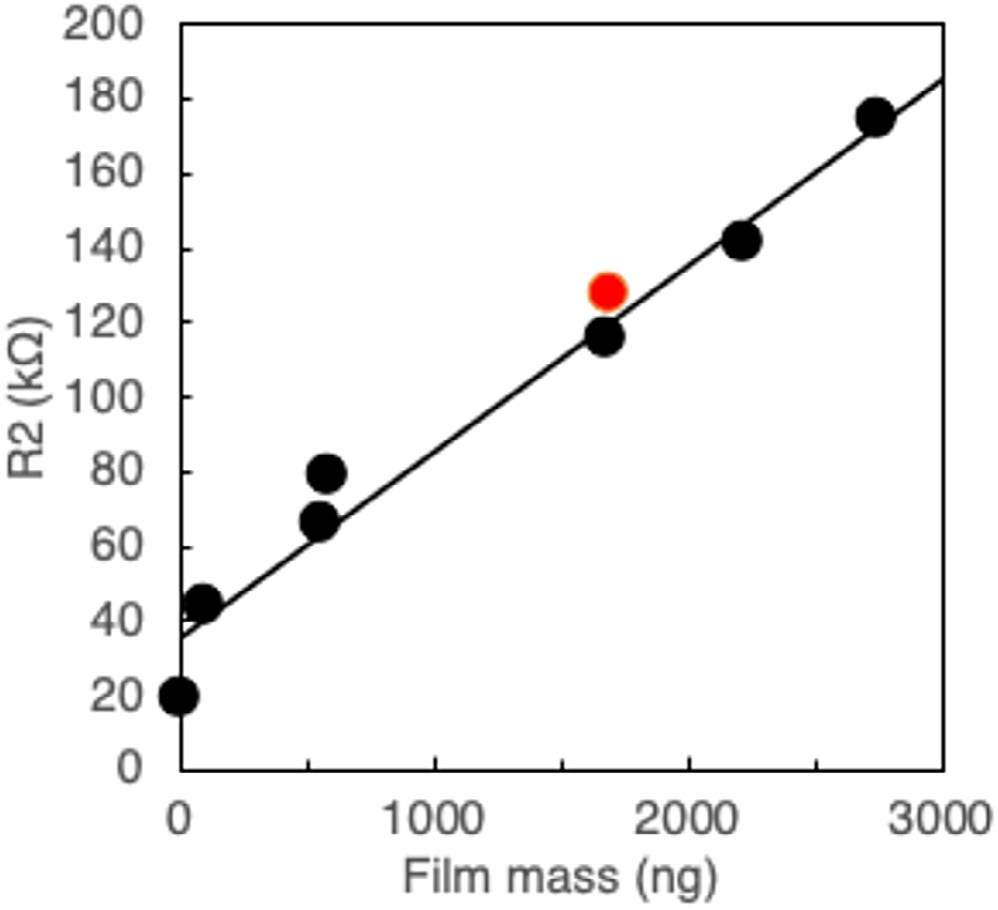
Relationship between PHBH film mass and *R*_2_ value. Black circles: without degradation; red circle: after 24 h degradation.

The charge transfer resistance *R*_2_ at the electrode interface generally increased with the thickness of the insulating layer on the electrode; therefore, the *R*_2_ value was also expected to increase in proportion to the mass of the PHBH film. *R*_2_ (the resistance of the PHBH film layer) exhibited a linear response to PHBH film mass (Figure 4). After degradation, the mass of the residual PHBH film was 1700 ng and the *R*_2_ value (red circle, Figure 4) was on the fitted line, suggesting that *R*_2_ depends only on film mass and is independent of film roughness caused by partial degradation. In contrast to *R*_1_ and *R*_2_, *n* reflects surface roughness. The unfabricated substrate exhibited an *n* of 0.913, whereas the pre‐degradation PHBH‐fabricated substrate exhibited an *n* of 0.830–0.876. The lower *n* of the PHBH film‐fabricated substrate was likely due to microscopic surface irregularities introduced during film fabrication. In contrast, the PHBH‐fabricated substrate's *n* was significantly lower after degradation, at 0.798, indicating that degradation significantly roughened the film's surface. Regarding the *Q* values of CPE, the *Q* value of the PHBH film substrate after degradation (film mass: 1700 ng) was 10.2 μF, which was greater than that (7.31 μF) of the undegraded PHBH film substrate having approximately the same mass (film mass: 1686 ng). Considering the decrease in *n* value of the film substrate after degradation, it is likely that the partial degradation of the film led to the formation of cracks and pinholes, which facilitated the penetration of water molecules, resulting in substantial charge accumulation owing to adsorption and desorption of the water molecules at the exposed gold electrode interface. These results also suggest that *C. testosteroni* caused local degradation of the PHBH film.

### 2.4 SECM Observation of PHBH Microbial Degradation

**FIGURE 5:**
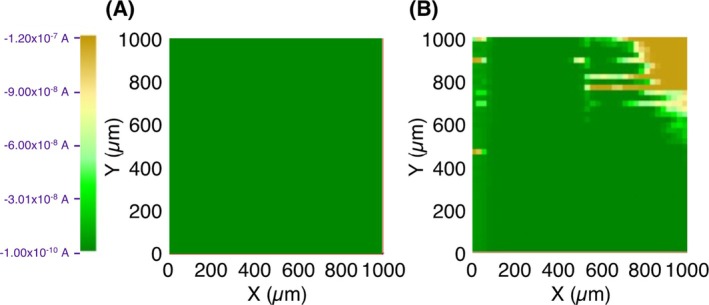
SECM images of PHBH film electrode before (A) and after 24 h microbial degradation (B).

The CV and impedance measurements suggested partial and localized microbial degradation of the PHBH film. However, in these measurements, the average current over the entire PHBH film was used to obtain the charge and equivalent circuit parameters. To complement these results, SECM was used to visually observe the local state of partial degradation of the PHBH film and the partial exposure of the gold surface. First, SECM measurements were performed on the PHBH film pre‐degradation substrate. A PHBH film (3000 ng) was fabricated on a quartz crystal gold electrode and used as the substrate electrode. The film surface (1 mm^2^) was scanned while measuring the viologen‐reduction current generated at the probe electrode, and the acquired current values were plotted (Figure 5A): only ca. 0.1 nA of current (green area) was generated over the entire 1 mm^2^ area, suggesting that the insulating nature of the PHBH film prevented the diffusion of the electron carrier on the film surface and reduced the current flow at the probe electrode. This suggests that the pre‐degradation PHBH film was sufficiently thick over the entire surface to prevent current flow and that no gold substrate was exposed.

After SECM measurement of the pre‐degradation substrate, the substrate was immersed in mineral medium, and its degradation was initiated by adding a bacterial suspension. The degradation reaction was conducted at 34°C for 24 h. After degradation, the electrode was used for SECM measurements (Figure 5B), in which a large proportion of the area remains green (representing the insulated regions), indicating that much of the PHBH film was not degraded. In some regions (in ochre, Figure 5B), the current was 100–1000 times higher than in the insulated regions, indicating the exposure of the gold surface and a cycle of electron carrier reduction at the probe electrode and oxidation of the substrate electrode surface, followed again by reduction at the probe electrode. SECM visually confirmed that the PHBH film was partially degraded by *C. testosteroni*, causing the partial exposure of the gold electrode surface, consistent with the ca. 10% degradation of the PHBH film revealed by QCM (red line, Figure 1A). Microbial and enzymatic degradation of biodegradable plastics is affected by the properties of the plastic, including their side chain length, surface hydrophobicity, and crystallinity [46, 47, 48, 49]. Considering our findings and the prior evidence, we assume that the structural properties of the PHBH film, such as the heterogeneous distribution of its component units and crystalline or amorphous structure, may cause local adsorption of the degrading bacteria and enzymes onto the PHBH film as well as local degradation by the secreted enzymes.

## 3 Conclusion

This study examined the mechanisms of microbial degradation of PHBH using a highly sensitive QCM in combination with electrochemical methods (CV, impedance analysis, and SECM). This enabled real‐time monitoring of bacterial adsorption to the PHBH film surface and of PHBH degradation at the cellular level. This combined analytical system, which is highly quantitative and achieves high spatiotemporal resolution, overcomes problems associated with the conventional methods for measuring biodegradable plastic degradation, including those associated with AFM and SEM. This system, which can be applied to any plastic that can be placed as a film on a QCM substrate, enables the assessment of plastic degradation at the cellular level—by degrading bacteria, their secreted enzymes, and components present in environmental samples. It therefore facilitates the comprehensive examination of the biodegradation—enzymatic, microbial, and environmental—of PHBH, other biodegradable plastics, and plastics discovered or synthesized in the future.

## 4 Experimental Section

### 4.1 Materials and Bacterium

The chemicals, of biochemical grade or the highest purity, were purchased from Sigma‐Aldrich (St Louis, MO, USA), TCI (Tokyo, Japan), or Wako Chemicals (Richmond, VA, USA). Commercial grade PHBH (containing 11 mol% 3HH) was donated by Kaneka Co. (Osaka, Japan). *C. testosteroni* YM1004, a PHA‐degrading bacterium isolated from seawater [50] and used here for PHBH degradation, was obtained from the Japan Collection of Microorganisms (JCM, Ibaraki, Japan).

### 4.2 QCM Apparatus

**FIGURE 6:**
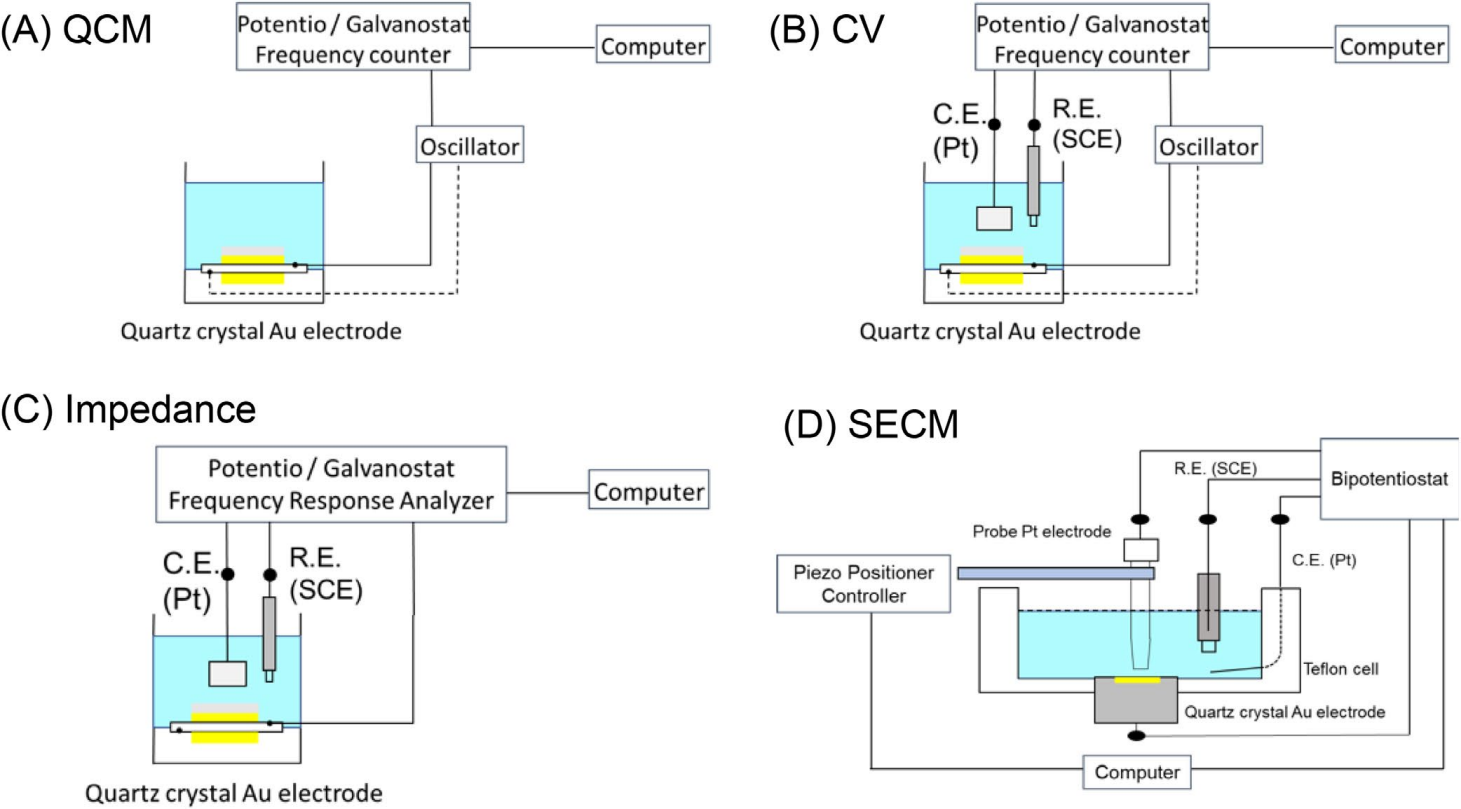
Schematic illustration of the experimental apparatuses used in this study. (A) QCM, (B) CV, (C) impedance and (D) SECM. CE, counter electrode; RE, reference electrode.

QCM experiments were performed using an EQCM analyzer (ALS/CHI 420A, BAS Inc., Tokyo, Japan) (Figure 6A). Using angle‐temperature (AT)‐cut quartz crystals with a 7.995 MHz fundamental frequency, a net decline in frequency of 1.0 Hz corresponds to a mass loss of 1.34 ng onto a gold evaporating crystal surface of area 0.196 cm^2^. The roughness factor of the gold surface was ca. 3.2. The experimental resolution for measurement of small frequency changes was 0.1 Hz, determined mainly by the oscillation stability of the crystal/oscillator combination. Temperature was controlled to within 0.05°C using a Cool Block Bath EC‐40R (As One Corporation, Osaka, Japan). No mathematical averaging or smoothing was applied to the QCM or voltammetry data. The sampling interval for QCM measurements was 2 ms.

### 4.3 PHBH Film Preparation

The EQCM substrate was rinsed with Milli‐Q water and dried under N_2_. The oscillator was washed with a freshly prepared 10% HNO_3_ solution, rinsed thoroughly with Milli‐Q water, and dried under N_2_. The resultant oscillator was washed with ethanol, rinsed thoroughly with Milli‐Q water, and dried under N_2_. A PHBH–chloroform solution (100 μL) was cast on one side of the oscillator, and the film was melted at 150°C for 1 h. The mass of the PHBH film was calculated using the Sauerbrey equation by measuring the change in quartz crystal frequency due to PHBH film formation. For example, when the film mass was 2000 ng, the thickness of the PHBH film on the substrate was estimated to be approximately 85 nm, based on a PHBH density of 1.20 g cm^−3^ [51] and an electrode area of 0.196 cm^2^.

### 4.4 QCM Measurement of PHBH Degradation by *C. testosteroni*

*C. testosteroni* was cultivated in 100 mL of LB medium (1% bacto‐tryptone, 0.5% yeast extract, and 0.5% NaCl, pH 7.0) at 30°C and 130 rpm for 18 h, to an OD_600_ of 2.5. The cells were harvested by centrifugation at 5000 × *g* and 4°C for 10 min. The pelleted cells were washed with the mineral medium, centrifuged again, and resuspended in mineral medium for PHBH degradation. The composition of mineral medium (pH 7.0) was as follows (per liter); 4.60 g KH_2_PO_4_, 11.60 g NaHPO_4_·12H_2_O, 1.00 g NH_4_Cl, 0.244 g MgSO_4_, 0.0336 g CaCl_2_·2H_2_O, and 0.1 g FeCl_3_·6H_2_O. The concentration of the bacterial suspension was calculated as 1 OD = 8 × 10^8^ cells mL^−1^ [52]. After QCM stabilization in 2.5 mL of mineral medium, microbial degradation was initiated by adding a bacterial suspension (0.5 mL) to the QCM cells. Microbial degradation of PHBH was performed at 34°C, and the change in frequency during the reaction was recorded using an EQCM analyzer (ALS/CHI 420A).

### 4.5 CV Measurement

An EQCM analyzer was used to measure CV (Figure 6B). A quartz crystal gold electrode and saturated calomel electrode (SCE) were purchased from BAS Inc. and used as the working and reference electrodes, respectively. The counter electrode was self‐made, using a platinum wire. The CV experimental potentials were standardized using a standard hydrogen electrode (SHE). CV measurements were performed at 25°C in 10 mM phosphate buffer (pH 7.0) containing 100 mM KCl.

### 4.6 Impedance Measurement

A quartz crystal gold electrode and an SCE were used as the working and reference electrodes, respectively (Figure 6C). The counter electrode was made of platinum wire. The potentiostat, PalmSens4 (PalmSens, Houten, Netherlands), controlled by PsTrace5 software, was used. The reaction temperature was controlled using a cool block bath (EC‐40R). Impedance measurements were performed at 25°C in 10 mM phosphate buffer (pH 7.0) containing 100 mM KCl. To measure impedance, a sinusoidal voltage of 5–10 mV was applied to the working electrode, and the frequency was varied from 1000 to 0.1 Hz (65 times). An EIS Spectrum Analyzer (PalmSens) was used to fit the impedance values to the equivalent circuit model curves, using Nyquist plots.

### 4.7 SECM Observation

SECM was performed using an ALS 920 scanning electrochemical microscope (CH Instruments, TX, USA) (Figure 6D). A platinum ultramicroelectrode probe (diameter: 10 μm) and SCE were used as the working and reference electrodes, respectively. A quartz crystal gold electrode was used as the substrate electrode, and a PHBH thin film was prepared. To determine the distance between the probe electrode and the substrate, a probe‐approach curve was generated. The approach measurements were carried out at 25°C in 10 mM phosphate buffer (pH 7.0) containing 1 mM amino viologen as an electron‐transfer agent and 100 mM KCl as an electrolyte, setting the probe electrode potential at −0.559 V and the substrate electrode potential at −0.259 V. When the current of the probe electrode reached 75% of the initial approach value, the probe was considered to be sufficiently close to the substrate, and surface imaging was performed with the probe depth direction fixed at this position. Surface scanning was performed by applying −0.559 V to the probe electrode and −0.259 V to the substrate electrode while measuring the viologen‐reduction current generated at the probe electrode. The scanning area was 1 mm^2^ on the film surface, with a resolution of 25 μm × 25 μm and a measuring time of 0.3 s for each point.

## Conflicts of Interest

The authors declare no conflicts of interest.

## Data Availability

The data that support the findings of this study are available from the corresponding author upon reasonable request.

## References

[1] Plastics Europe , “Plastics—The Fast Facts 2024,” accessed March 2025, https://plasticseurope.org/wp‐content/uploads/2024/11/PE_TheFacts_24_digital‐1pager.pdf.

[2] R. Geyer , J. R. Jambeck , and K. L. Law , “Production, Use, and Fate of All Plastics Ever Made,” Science Advances 3 (2017): e1700782, 10.1126/sciadv.1700782. 28776036 PMC5517107

[3] W. W. Y. Lau , Y. Shiran , R. M. Bailey , et al., “Evaluating Scenarios Toward Zero Plastic Pollution,” Science 369 (2020): 1455–1461, 10.1126/science.aba9475. 32703909

[4] A. Bher , P. C. Mayekar , R. A. Auras , and C. E. Schvezov , “Biodegradation of Biodegradable Polymers in Mesophilic Aerobic Environments,” International Journal of Molecular Sciences 23 (2022): 12165, 10.3390/ijms232012165. 36293023 PMC9603655

[5] B. Dalton , P. Bhagabati , J. De Micco , R. B. Padamati , and K. O'Connor , “A Review on Biological Synthesis of the Biodegradable Polymers Polyhydroxyalkanoates and the Development of Multiple Applications,” Catalysts 12, no. 3 (2022): 319, 10.3390/catal12030319.

[6] Kaneka Corporation , “How Does KANEKA Biodegradable Polymer Green Planet Make the World Healthy?,” accessed March 2025, https://www.kaneka.co.jp/en/solutions/phbh/index.html.

[7] N. Hyodo , H. Gan , M. Ilangovan , et al., “Coastal and Deep‐Sea Biodegradation of Polyhydroxyalkanoate Microbeads,” Scientific Reports 14 (2024): 10302, 10.1038/s41598-024-60949-z. 38705904 PMC11070421

[8] F. Ruggero , R. Gori , and C. Lubello , “Methodologies to Assess Biodegradation of Bioplastics During Aerobic Composting and Anaerobic Digestion: A Review,” Waste Management and Research 37 (2019): 959–975, 10.1177/0734242X19854127. 31218932

[9] J. R. A. Pires , V. G. L. Souza , P. Fuciños , L. Pastrana , and A. L. Fernando , “Methodologies to Assess the Biodegradability of Bio‐Based Polymers‐Current Knowledge and Existing Gaps,” Polymers 14 (2022): 1359, 10.3390/polym14071359. 35406232 PMC9002992

[10] Y. Kikkawa , T. Hirota , K. Numata , et al., “In‐Situ Atomic Force Microscopy Observation of Enzymatic Degradation in Poly(Hydroxyalkanoic Acid) Thin Films: Normal and Constrained Conditions,” Macromolecular Bioscience 4 (2004): 276–285, 10.1002/mabi.200300065. 15468218

[11] H. Sashiwa , R. Fukuda , T. Okura , S. Sato , and A. Nakayama , “Microbial Degradation Behavior in Seawater of Polyester Blends Containing Poly(3‐Hydroxybutyrate‐Co‐3‐Hydroxyhexanoate) (PHBHHx),” Marine Drugs 16 (2018): 34, 10.3390/md16010034. 29342118 PMC5793082

[12] C. Kato , A. Honma , S. Sato , T. Okura , R. Fukuda , and Y. Nogi , “Poly 3‐Hydroxybutyrate‐Co‐3‐Hydroxyhexanoate Films Can Be Degraded by the Deep‐Sea Microbes at High Pressure and Low Temperature Conditions,” High Pressure Research 39 (2019): 248–257, 10.1080/08957959.2019.1584196.

[13] K. Yamashita , Y. Aoyagi , H. Abe , and Y. Doi , “Analysis of Adsorption Function of Polyhydroxybutyrate Depolymerase From *Alcaligenes faecalis* T1 by Using a Quartz Crystal Microbalance,” Biomacromolecules 2 (2001): 25–28, 10.1021/bm0000844. 11749150

[14] K. Yamashita , T. Funato , Y. Suzuki , S. Teramachi , and Y. Doi , “Characteristic Interactions Between Poly(Hydroxybutyrate) Depolymerase and Poly[(*R*)‐3‐Hydroxybutyrate] Film Studied by a Quartz Crystal Microbalance,” Macromolecular Bioscience 3 (2003): 694–702, 10.1002/mabi.200300004.

[15] K. Kurokawa , K. Yamashita , Y. Doi , and H. Abe , “Surface Properties and Enzymatic Degradation of End‐Capped Poly(l‐Lactide),” Polymer Degradation and Stability 91 (2006): 1300–1310, 10.1016/j.polymdegradstab.2005.08.015.

[16] Y. Hou , J. Chen , P. Sun , Z. Gan , and G. Zhang , “In Situ Investigations on Enzymatic Degradation of Poly(ɛ‐Caprolactone),” Polymer 48 (2007): 6348–6353, 10.1016/j.polymer.2007.08.054.

[17] S. D. Sommerfeld , Z. Zhang , M. C. Costache , S. L. Vega , and J. Kohn , “Enzymatic Surface Erosion of High Tensile Strength Polycarbonates Based on Natural Phenols,” Biomacromolecules 15 (2014): 830–836, 10.1021/bm4016539. 24432806 PMC3983148

[18] V. Perz , M. T. Zumstein , M. Sander , S. Zitzenbacher , D. Ribitsch , and G. M. Guebitz , “Biomimetic Approach to Enhance Enzymatic Hydrolysis of the Synthetic Polyester Poly(1,4‐Butylene Adipate): Fusing Binding Modules to Esterases,” Biomacromolecules 16 (2015): 3889–3896, 10.1021/acs.biomac.5b01219. 26566664

[19] M. T. Zumstein , H.‐P. E. Kohler , K. McNeill , and M. Sander , “Enzymatic Hydrolysis of Polyester Thin Films: Real‐Time Analysis of Film Mass Changes and Dissipation Dynamics,” Environmental Science & Technology 50 (2016): 197–206, 10.1021/acs.est.5b04103. 26599203

[20] Z. Dong , P. Zhang , S. Kralj , Y. Ji , and U. Schwaneberg , “Synergism of Endo and Exo‐α‐1,3‐Glucanases in α‐1,3‐Glucan Degradation: A Kinetic Study,” ACS Sustainable Chemistry & Engineering 12 (2024): 9123–9132, 10.1021/acssuschemeng.4c01469.

[21] Z. Zhou , Y. Wen , Q. Liao , et al., “Evaluation of the Assembly Process and Green Marine Antifouling Potential of Quaternary Ammonium Chitosan/κ‐Carrageenan Polyelectrolyte Multilayers Using Quartz Crystal Microbalance,” ACS Sustainable Chemistry & Engineering 13 (2025): 7017–7033, 10.1021/acssuschemeng.5c00213.

[22] H. Wang , T. Zhang , K. Chen , L. Long , and S. Ding , “Biochemical Characterization and Polyester‐Binding/Degrading Capability of Two Cutinases From *Aspergillus fumigatus* ,” Microorganisms 13 (2025): 1121, 10.3390/microorganisms13051121. 40431293 PMC12114444

[23] G. Sauerbrey , “Verwendung von Schwingquarzen zur Wägung dünner Schichten und zur Mikrowägung,” Zeitschrift für Physik 155 (1959): 206–222.

[24] N. Asakura , T. Kamachi , and I. Okura , “Direct Monitoring of the Electron Pool Effect of Cytochrome *c* _3_ by Highly Sensitive EQCM Measurements,” JBIC Journal of Biological Inorganic Chemistry 9 (2004): 1007–1016, 10.1007/s00775-004-0604-6. 15517437

[25] E. Kobayashi , Y. Hirose , T. Kamachi , K. Tabata , I. Okura , and N. Asakura , “Investigation of the Key Heme in Cytchrome *c* _3_ to the Electron Pool Effect by Highly Sensitive EQCM Technique,” Electrochemistry 80 (2012): 312–314, 10.5796/electrochemistry.80.312.

[26] L. F. Q. P. Marchesi , F. R. Simões , L. A. Pocrifka , and E. C. Pereira , “Investigation of Polypyrrole Degradation Using Electrochemical Impedance Spectroscopy,” Journal of Physical Chemistry B 115 (2011): 9570–9575, 10.1021/jp2041263. 21721565

[27] G. W. Walter , “Application of Impedance Measurements to Study Performance of Painted Metals in Aggressive Solutions,” Journal of Electroanalytical Chemistry 118 (1981): 259–273, 10.1016/S0022-0728(81)80546-3.

[28] F. Mansfeld , M. W. Kendig , and S. Tsai , “Evaluation of Corrosion Behavior of Coated Metals With AC Impedance Measurements,” Corrosion 38 (1982): 478–485, 10.5006/1.3577363.

[29] A. Sabot and S. Krause , “Simultaneous Quartz Crystal Microbalance Impedance and Electrochemical Impedance Measurements. Investigation Into the Degradation of Thin Polymer Films,” Analytical Chemistry 74 (2002): 3304–3311, 10.1021/ac0200724. 12139033

[30] D. T. Pierce and A. J. Bard , “Scanning Electrochemical Microscopy. 23. Retention Localization of Artificially Patterned and Tissue‐Bound Enzymes,” Analytical Chemistry 65 (1993): 3598–3604, 10.1021/ac00072a012. 8311246

[31] J. J. Santana , J. Izquierdo , and R. M. Souto , “Uses of Scanning Electrochemical Microscopy (SECM) for the Characterization With Spatial and Chemical Resolution of Thin Surface Layers and Coating Systems Applied on Metals: A Review,” Coatings 12 (2022): 637, 10.3390/coatings12050637.

[32] J. Wegener , A. Janshoff , and H. J. Galla , “Cell Adhesion Monitoring Using a Quartz Crystal Microbalance: Comparative Analysis of Different Mammalian Cell Lines,” European Biophysics Journal 28 (1999): 26–37, 10.1007/s002490050180. 9933921

[33] R. S. Friedlander , N. Vogel , and J. Aizenberg , “Role of Flagella in Adhesion of *Escherichia coli* to Abiotic Surfaces,” Langmuir 31 (2015): 6137–6144, 10.1021/acs.langmuir.5b00815. 25945399

[34] A. Fulgione , M. Cimafonte , B. D. Ventura , et al., “QCM‐Based Immunosensor for Rapid Detection of *Salmonella* Typhimurium in Food,” Scientific Reports 8 (2018): 16137, 10.1038/s41598-018-34285-y. 30382128 PMC6208438

[35] G. Bratbak and I. Dundas , “Bacterial Dry Matter Content and Biomass Estimations,” Applied and Environmental Microbiology 48 (1984): 755–757, 10.1128/aem.48.4.755-757.1984. 6508285 PMC241608

[36] Z. Kerner and T. Pajkossy , “Impedance of Rough Capacitive Electrodes: The Role of Surface Disorder,” Journal of Electroanalytical Chemistry 448 (1998): 139–142, 10.1016/S0022-0728(98)00025-4.

[37] A. Lasia , “Impedance of Porous Electrodes,” Journal of Electroanalytical Chemistry 397 (1995): 27–33, 10.1016/0022-0728(95)04177-5.

[38] L. Nyikos and T. Pajkossy , “Fractal Dimension and Fractional Power Frequency‐Dependent Impedance of Blocking Electrodes,” Electrochimica Acta 30 (1985): 1533–1540, 10.1016/0013-4686(85)80016-5.

[39] S. T. Amaral and I. L. Muller , “Effect of Silicate on Passive Films Anodically Formed on Iron in Alkaline Solution as Studied by Electrochemical Impedance Spectroscopy,” Corrosion 55 (1999): 17–23, 10.5006/1.3283960.

[40] J. B. Jorcin , M. E. Orazem , N. Pébère , and B. Tribollet , “CPE Analysis by Local Electrochemical Impedance Spectroscopy,” Electrochimica Acta 51 (2006): 1473–1479, 10.1016/j.electacta.2005.02.128.

[41] P. Zoltowski , “On the Electrical Capacitance of Interfaces Exhibiting Constant Phase Element Behaviour,” Journal of Electroanalytical Chemistry 443 (1998): 149–154, 10.1016/S0022-0728(97)00490-7.

[42] T. Pajkossy , “Impedance of Rough Capacitive Electrodes,” Journal of Electroanalytical Chemistry 364 (1994): 111–125, 10.1016/0022-0728(93)02949-I.

[43] J. T. Zhang , J. M. Hu , J. Q. Zhang , and C. N. Cao , “Studies of Water Transport Behavior and Impedance Models of Epoxy‐Coated Metals in NaCl Solution by EIS,” Progress in Organic Coatings 51 (2004): 145–151, 10.1016/j.porgcoat.2004.08.001.

[44] C. Zhu , R. Xie , J. Xue , and L. Song , “Studies of the Impedance Models and Water Transport Behaviors of Cathodically Polarized Coating,” Electrochimica Acta 56 (2011): 5828–5835, 10.1016/j.electacta.2011.04.068.

[45] P. L. Bonora , F. Deflorian , and L. Fedrizzi , “Electrochemical Impedance Spectroscopy as a Tool for Investigating Underpaint Corrosion,” Electrochimica Acta 41 (1996): 1073–1082, 10.1016/0013-4686(95)00440-8.

[46] H. Yang , G. Chen , and J. Wang , “Microplastics in the Marine Environment: Sources, Fates, Impacts and Microbial Degradation,” Toxics 9 (2021): 41, 10.3390/toxics9020041. 33671786 PMC7927104

[47] H. Abe and Y. Doi , “Structural Effects on Enzymatic Degradabilities for Poly[(*R*)‐3‐Hydroxybutyric Acid] and Its Copolymers,” International Journal of Biological Macromolecules 25 (1999): 185–192, 10.1016/S0141-8130(99)00033-1. 10416666

[48] Z. Li , H. Lin , N. Ishii , G.‐Q. Chen , and Y. Inoue , “Study of Enzymatic Degradation of Microbial Copolyesters Consisting of 3‐Hydroxybutyrate and Medium‐Chain‐Length 3‐Hydroxyalkanoates,” Polymer Degradation and Stability 92 (2007): 1708–1714, 10.1016/j.polymdegradstab.2007.06.001.

[49] K. Min , J. D. Cuiffi , and R. T. Mathers , “Ranking Environmental Degradation Trends of Plastic Marine Debris Based on Physical Properties and Molecular Structure,” Nature Communications 11 (2020): 727, 10.1038/s41467-020-14538-z. PMC7002677 32024839

[50] K. Mukai , K. Yamada , and Y. Doi , “Enzymatic Degradation of Poly(Hydroxyalkanoates) by a Marine Bacterium,” Polymer Degradation and Stability 41 (1993): 85–91, 10.1016/0141-3910(93)90066-R.

[51] MatWeb , “MatWeb, Material Property Data,” accessed March 2025, https://www.matweb.com/index.aspx.

[52] B. R. Stenzler , R. Zhang , J. D. Semrau , A. A. DiSpirito , and A. J. Poulain , “Diffusion of H_2_S From Anaerobic Thiolated Ligand Biodegradation Rapidly Generates Bioavailable Mercury,” Environmental Microbiology 24 (2022): 3212–3228, 10.1111/1462-2920.16078. 35621051

